# Engraving of stainless-steel wires to improve optical quality of closed-loop wire-guided flow jet systems for optical and X-ray spectroscopy

**DOI:** 10.3389/fmolb.2023.1079029

**Published:** 2023-06-14

**Authors:** Alessandra Picchiotti, Martin Precek, Anna Zymaková, Tim Erichlandwehr, Yingliang Liu, Tuomas Wiste, Petr Kahan, Irene Fernandez-Cuesta, Jakob Andreasson

**Affiliations:** ^1^ ELI Beamlines Facility, The Extreme Light Infrastructure ERIC, Dolni Brezany, Czechia; ^2^ The Hamburg Centre for Ultrafast Imaging, Hamburg, Germany; ^3^ Department of Physics, Universität Hamburg, Hamburg, Germany; ^4^ Deutsches Elektronen-Synchrotron, Hamburg, Germany; ^5^ Institute of Biotechnology, Czech Academy of Sciences, Vestec, Czechia; ^6^ Institute of Physics, Czech Academy of Sciences, Prague, Czechia

**Keywords:** microfluidics, spectroscopy, etching, engraving, wet-etching, jet, x-ray, laser

## Abstract

This paper describes performance enhancement developments to a closed-loop pump-driven wire-guided flow jet (WGJ) for ultrafast X-ray spectroscopy of liquid samples. Achievements include dramatically improved sample surface quality and reduced equipment footprint from 7 × 20 cm^2^ to 6 × 6 cm^2^, cost, and manufacturing time. Qualitative and quantitative measurements show that micro-scale wire surface modification yields significant improvements to the topography of the sample liquid surface. By manipulating their wettability, it is possible to better control the liquid sheet thickness and to obtain a smooth liquid sample surface, as demonstrated in this work.

## Introduction

The liquid and soft states of matter offer unique opportunities to study a range of chemical and biochemical reactions (Galinis, 2017), and recent developments in ultrafast techniques for study of process dynamics have drawn the attention of scientists worldwide. Hence, in time-resolved spectroscopy, there is an ever-growing need for liquid sample delivery systems, as study and application of photon science continue to expand. Experiments at table-top instruments as well as large facilities, such as X-ray free-electron lasers (XFEL), pose unique challenges on all aspects of experiment realization, from sample preparation to sample delivery, and finally data acquisition and analysis. New developments in XFEL science broaden research possibilities in time-resolved spectroscopy, thanks to the high brilliance and coherence of XFEL sources. The latest achievements in XFEL technology and science enable experiments that require short pulse duration with a temporal resolution down to the femtosecond range (Duris, 2020).

To avoid effects of radiation damage affecting X-ray spectroscopy or laser spectroscopy measurements, the sample often needs to be refreshed between exposures (Fondell, 2017), making conventional free-standing sample delivery systems unsuitable. Furthermore, the ultrashort duration of XFEL pulses allows spectra and diffraction patterns to be collected before sample destruction (Chapman, 2014), even at room temperature. Thus, it is crucial to conceive systems that deliver a flow of sample at a rate that is high enough for each shot to expose a fresh sample volume. At the same time, sample consumption must be minimized for samples which are expensive or laborious to acquire or produce. Minimization of the total volume of sample needed for an experiment goes hand in hand with reducing the overall footprint of the setup. Space is typically at a premium and footprint can be a decisive factor in the practicality of an experimental setup. Experiments performed at user facilities can also require setups that are portable, and compatible with travel.

As absorption cross sections in the X-ray and optical regimes vary by orders of magnitude, a high degree of adaptability concerning the thickness of the liquid layer is required from a setup that is intended to be used in both X-ray and optical spectroscopy, or in optical/X-ray pump-probe experiments. Furthermore, a flat liquid surface is preferred to maintain good optical quality and limit degradation of data quality, particularly in the optical range. The sample delivery system must be optimized to avoid undue background noise or distortions to the data that could mask the true weak spectroscopic signals from the sample while delivering a stable liquid sheet (Kim, 2016). While satisfying all the constraints outlined above, systems for sample delivery should also ideally be technically undemanding, so it can be operated by non-expert scientists or technicians at research facilities and table-top spectroscopy labs.

Many flow-based delivery systems for liquid samples have been developed (Ekimova, 2015, Galinis, 2017; Weierstall, 2014; Tauber, 2003; Menzi, 2020) for a variety of different applications, following their specific requirements (Grunbein, 2019). An early system developed by one of the authors (Picchiotti, 2015) fulfilled many of the requirements outlined above. The closed-loop, wire-guided jet system (WGJ) was relatively compact, versatile, standalone, and able to fit in a typical optical spectroscopy setup. The first version handled sample volumes down to 0.5 mL, with a sufficient flow rate to support 4 kHz laser systems. By adjusting the flow rate, it was possible to control the thickness of the liquid sheet between 100 μm and 400 μm or more, depending on the gear pump employed, while ensuring its overall fluid dynamic and operational stability with less than 1% fluctuation in optical stability. Absorbance changes in the samples on the level of 100 μOD were measured by the authors in a previous laboratory configuration. The short- and long-term stability allowed the successful acquisition of spectra with low signal-to-noise ratios of fragile samples, both in the visible and in the x-ray range (Prokhorenko, 2016; Pour, 2017; Stange, 2018; Oppermann, 2019; Picchiotti, 2019; Cannelli, 2019; Pastorczak, 2019).

At the ELI-Beamlines facility, a compact laser-driven [Zymakova 2021] water-jet plasma X-ray source (PXS) (MiajaAvila, 2015; Zymaková, 2020) is available with an X-ray spectroscopy setup [Zymakova 2022] which uses von Hamos geometry (Szlachetko, 2012). The water-jet PXS generates a pulsed broadband X-ray beam that, in combination with an optical beam, can be used for time-resolved spectroscopic studies. In this type of studies, shot-to-shot refreshment of the sample is required to reduce the effects of sample damage, and a container-free delivery system is preferred to remove the contribution from the container (e.g., a cuvette wall). A compact closed-loop liquid sample delivery system with low sample consumption is an ideal solution for the setup. Other flow systems require much more sample (dozens of milliliters at least), while the present one can be run with sub-milliliter volumes. This is one of the main advantages of the technology presented in (Picchiotti, 2015) and in the upgraded version presented in this paper as compared to other systems: since the WGJ is a low-pressure liquid system, it is possible to minimize the used sample to sub-milliliters volumes, which is essential for biologically relevant samples. A very popular liquid flow system among spectroscopists is the super-thin (Galinis, 2017; Menzi, 2020; Barnard, 2022), which works in a completely different regime than the one presented here. Those other systems rely on very small nozzles and work in the high-pressure regime. This means that the noozles clog very frequently, they are not usable for big molecular systems like proteins, and need an HPLC pump. Despite those systems work reliably for certain experimental conditions and for a subset of chemicals and molecules, it is limited to inorganic or simple organic systems. Moreover, it requires xxx amount of space, including HPLC pumps and a total volume of the sample of at least hundreds of milliliters, absolutely not feasible for all the biologically relevant molecules and many expensive molecules in general.

What was left to improve in the previous iteration, firstly it was to minimize the overall space occupied by the jet, the dampener, the pump and other elements. For this purpose, we have developed further the WGJ (Picchiotti, 2015) to fit this and other X-ray set ups. In particular, the geometrical constraints of the X-ray station required a miniaturization of the system. Furthermore, requirements on rapid and multiple sample exchange at international user facilities (like ELI Beamlines) required the development of a concept for rapid and cost-effective manufacturing of parts as several parts of the jet system are replaced (or cleaned and maintained) during sample exchange. Secondly, as compared to the previous version, the WGJ shows a giant improvement on the optical quality, essential for experiments utilizing visible and ultraviolet light. This is a characteristic that other jet system developers are aiming at, for instance Galinis et al. (Galinis, 2017). To evaluate the optical quality, and in general to measure the thickness of the liquid, essential for spectroscopy experiments, a cost effective and reliable method for the characterization of the optical properties of the sample flow is needed to facilitate further developments of liquid jet/sheet systems, as well as for fast and efficient optimization of the sample delivery before and during experiment, especially in time-starving beam-time experiments at big facilities. Unluckily, the methods found in literature work only for thin leaflets, generated by high-pressure jets, while the one presented here can achieve thicker layers of liquid, which is needed in some experimental settings, e.g., when the cross section of the sample dissolved in the liquid is very small. Therefore, the reported method to characterize the optical quality of high pressure jets, interference, is not applicable here.

In the present short communication, we report two key upgrades to the WGJ system: 1) the miniaturization of the jet to fit in the workstation at ELI-Beamlines, and 2) an improved quality of the liquid film topology (surface). In short:

(1) The system has been miniaturized and simplified to address the geometrical constraints of the X-ray experiment, thus decreasing the footprint of the WGJ to render it more agile. The miniaturization results in a reduction of the system footprint from 7 × 20 cm^2^ down to 6 × 6 cm^2^. Some of the parts composing the jet have been produced by 3D printing, and stress-tested in operation when used to build the jet. This also results in a decrease of the manufacturing costs. Other liquid jet systems require HPLC pumps, which take much more space in a laboratory setting, and are heavier than the low-pressure gear pumps needed for the present WGJ.
(2) The protocol for the treatment of the stainless-steel wires described here, results in a surface modification of the wires themselves, which in turn allows for a control on the topology of the flowing film between the wires, and in general on its quality. This aspect is fundamental in key experiments that require an optical interaction between the liquid and a light beam, like spectroscopic experiments. No other low-pressure jet system has achieved the optical quality we present here in the liquid thickness range that the WGJ can operate (the liquid thickness from 500 to 50 μm was successfully tested in our laboratories). The characterization method proposed here, allows to measure the topology of the liquid and its thickness over the full liquid sheet area at the same time. This is a completely new method as compared to the method reported for example, in the paper of Menzi et al. among others [Menzi 2020], where interference pattern was measured.

Here, we describe the design and fabrication of a new WGJ jet design and its fabrication, fitting in the workstation at ELI-Beamlines. We also present a wet etching wire treatment, and the resulting change in the surface roughness, imaged by SEM. The associated changes in wettability are characterized by contact angle measurements in a similar flat sample, showing that the treatment results in an increase of the water contact angle. Moreover, we show how the treatment results in a smoother liquid curtain, observed by light scattering of an incident laser beam, and measured by quantitative acquisition of the fluorescence intensity along the liquid. In this way, the optical quality of the liquid is comparable to the one in a flow-cell, but without walls, pivotal in experiments where walls will introduce scattering, group velocity dispersion and other artifacts.

## Materials and methods

### Wire treatment (wet etching)

The liquid sheet of the WGJ jet is created in-between stainless-steel wires (AISI 316, purchased from Advent Research Materials, Ltd., already straight rather than coiled). Then, the wires were treated by wet etching. For this, the wires were left in a bath of hydrochloric acid (VWR, LLC) with a concentration of 37% at room temperature for 30 min. Afterwards, they were sonicated in water, rinsed with distilled water (Purist UV Ultrapure Water System), swept with optical tissues (Thorlabs, Inc.) and imbued in acetone (spectroscopic quality from VWR, LLC) until clean. In this process the hydrochloric acid solution takes on a pale-yellow color, indicating a non-negligible presence of Fe^3+^ dissolved from the surfaces of the stainless-steel wires, as ordinarily occurs when etching stainless steel with strong acids (Li, 2012).

### Scanning electron microscope images

We investigated the surface of the wires using a Thermo/FEI Quattro S scanning electron microscope in high vacuum mode using an Everhart-Thornley detector in secondary electron (SE) collection mode, with an accelerating voltage of 30 kV. These images are shown in Figures 3A-D.

### Contact angle measurements

The water contact angle (WCA) measurements were performed on a “Drop shape analyzer DSA25E″ by Krüss, in the ‘sessile drop’-configuration and averaged over 120 individual measurement points. The drop shape and contact angle was fit and measured automatically with the software in the instrument.

These measurements were performed on a rectangular block of the same stainless-steel alloy, with a flat surface, with an area of 10 × 20 mm^2^. This flat surface allows reproducibility and high accuracy quantification of the water contact angle, since the diameter of the wires employed in the WGJ (0.5 mm) are too small, and moreover, the surface of the wires is highly curved, which will prevent standard quantification. Prior to any treatment, the stainless-steel surface was fully immersed in acetone while being sonicated at ambient temperature for 120 s to remove surface contamination. The cleaning process was then repeated with isopropanol (IPA). Finally, the stainless-steel piece was wet etched with concentrated hydrochloric acid (37%) while being sonicated for a total time of 30 min at ambient temperature. In this way, we can reliably measure the contact angle before and after 30 min of wet etching. The results are shown in Figure 4.

### Light transmission through the flowing film by photographic imaging

**FIGURE 1:**
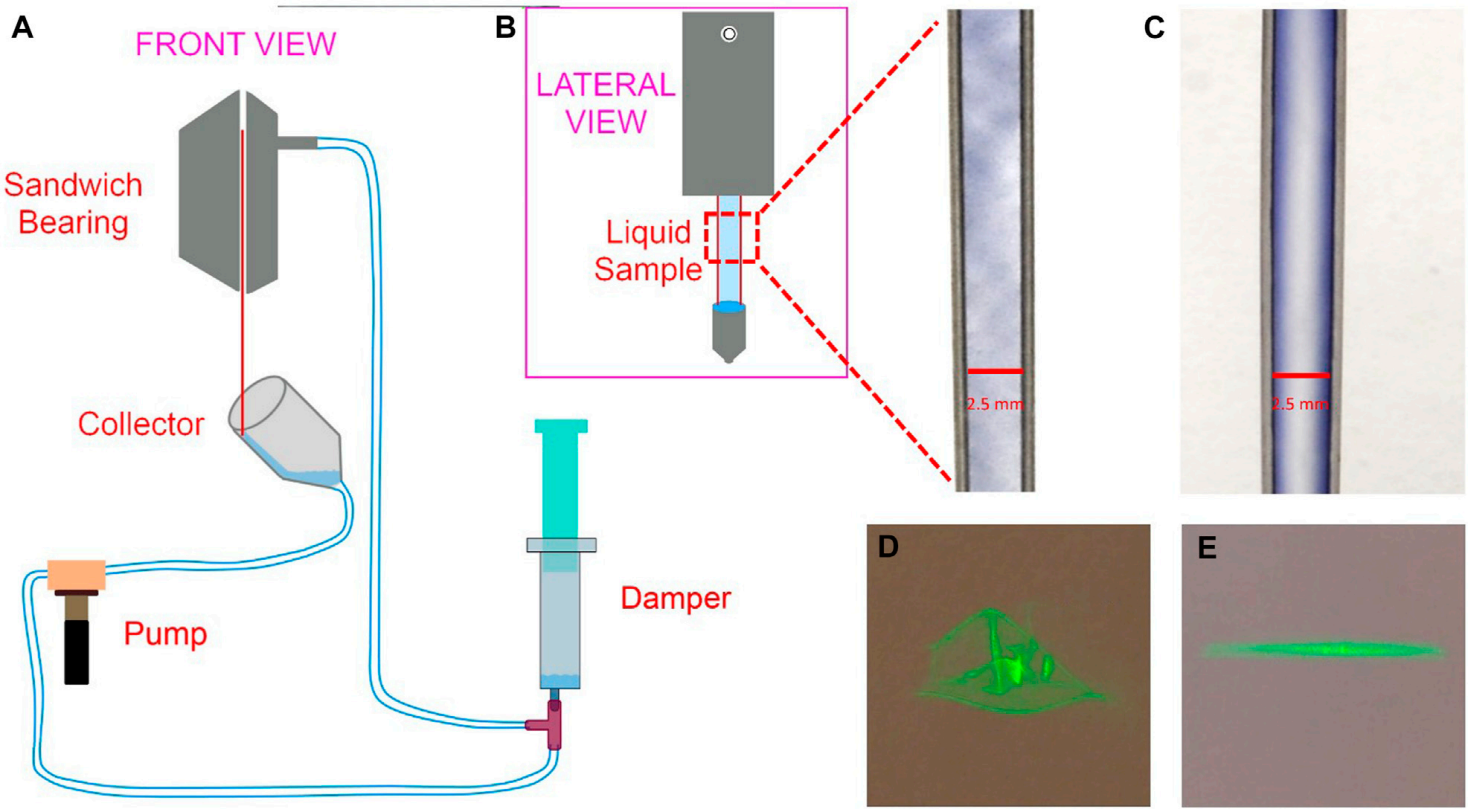
**(A)** A schematic of the WGJ system, with a front view of the sandwich bearing in the insert (top right). Wires are red and the liquid flowing between the wires, gathered by the collector on the bottom, is blue. Figure from (Picchiotti, 2015) **(B,C)** Photograph of a detail of the U-shaped wires, from the two WGJ systems used in this work, **(A)** WGJ with untreated wires mounted, and **(B)** WGJ with wires undergone the hydrochloric acid treatment. Nile Blue dissolved in distilled water was used in both cases to create the flowing film between the wires. Photos courtesy of Mullana (Lisa Schmidt) **(D,E)** Difference in the optical quality of transmitted LED light through the WGJs in **(B)** and **(C)**; **(D)** with untreated wires and **(E)** with treated wires mounted.

To test the improvement of the optical surface quality of the produced liquid film, two identical jets were built, one with un-treated wires and one with wires that had undergone wet etching with the hydrochloric acid. All other parameters were kept identical. A low power, continuous wave diode laser was used as a point-source to illuminate the running liquid film, pointing at a location of the film a few millimeters below the edge of the sandwich bearing. The light transmitted through the film was projected onto a white background and images of the projections were taken using normal photographic cameras, as the ones mounted on smartphones, using an exposure time of 1/10th seconds. The images are shown in Figure 1.

### Fluorescence measurements

A solution of 1 μg/mL concentration of Rhodamine 6G (Rh6G) (Sigma Aldrich) dissolved in 10% IPA and 90% de-ionized water (Purist UV Ultrapure Water System from Rephile Bioscience, Ltd.) was used as the circulating liquid sample for both jet systems, with treated (wet etched) and untreated wires mounted. The absorbance spectrum of Rhodamine 6G in water consists of a peak at about 530 nm and a shoulder around 500 nm, and the emission spectrum is a curve covering the wavelength range between 400 nm and 700 nm. This makes Rh6G a good dye for the LED we employed: the light of a blue LED with a wavelength of 455 nm (model M455L4, Thorlabs, Inc.) was used to illuminate the Rhodamine 6G solution flowing between the wires of the jet. The emitted fluorescence light from the flowing liquid film was then captured with a digital fluorescence microscope (model AM4115T-CFVW, Dino-Lite), with 1.3 Megapixel resolution. The images were recorded with a ×20 objective.

To quantify the fluorescence and correlate it to the liquid sheet thickness, the images were subsequently processed with ImageJ v1.50i. The RGB-formatted TIFF images produced by the digital microscope were separated into the three color-channels. Only the green channel was used in further processing, as it had the maximum signal due to the specific Rhodamine 6G fluorescence spectrum (Penzkofer, 1987). An image of a flat cuvette of 0.5 mm thickness, filled with the same Rhodamine 6G solution, was used as reference to calculate the absolute thickness of the liquid layer in the WGJ jet from the ratio of the emitted fluorescence intensity. This has the advantage of compensating for any inhomogeneity in the light field of the blue LED light source. The XY scale of the image, in microns/pixel, was set up via images using the known dimension of the cuvette surface. A region of interest (ROI) was cut out of the images of the jet fluorescence to enable easier data processing and visualization. The ROIs of both the treated and untreated wire setups were selected as the area where there was no reflection of the light from the wires, and where there was overlap with the fluorescence of the cuvette. These ROIs were then applied onto the reference cuvette image to create equivalent cutouts. All the selections were adjusted using rotations and distortions in order to create a rectangular grid. The images were subjected to image mathematical operations to calculate the ratio against the reference cuvette thickness, giving the absolute values of the thickness in both the *Z*-axis and the color scale. Figure 5 shows the results of the image processing described above for the sheets obtained with the jet with untreated wires (on the left) and for the one with treated ones (on the right). The difference in the *X*-axis, corresponding to the width of the liquid flow, is due to a different mounting at the moment of taking the images, which can differ by up to half a millimeter. These images were chosen as the most representative ones. The difference in the sheet width does not influence the comparative results reported here. They are shown in Figure 5.

## Results and discussion

### Building of the WGJ and including 3D printed parts

The WGJ includes a microfluidic pump, a collector, the sandwich block holding the wires, a damping syringe, and fluidic connections and connecting tubes. The sandwich block is home-built (Picchiotti, 2015; Laimgruber, 2006), and as in the previous version, a channel 0.2 mm in depth was milled in the sandwich block. The internal channel is milled symmetrically on both sides of the sandwich block. The width of the channel defines the spacing between the wires. The WGJ utilized for this work has two configurations, where the spacing (internal width from the two vertical wires) is 2.5 mm or 3.5 mm. The blocks are made from stainless steel AISI 316-L. The flow jet is created using stainless steel wires that guide the liquid between them. The wires are manually bent into a U-shape and have a length of 70–90 mm after bending.

Repeating the design of the previous version, the ends of the wires exiting from the sandwich block are positioned into the collector such that their tips nearly touch the internal wall of the collector to allow for the creation of a smooth curtain flow. However, to facilitate fast sample exchange, replacement parts need to be readily available and to reduce costs and manufacturing time, we 3D-print collectors in VeroClear (Stratasys, 2020) (Stratasys, Ltd.) using a polyjet printer (Objet 30 Pro). VeroClear is a transparent polyjet photopolymer that simulates clear acrylic, is well suited for biological solutions (Rimington, 2018) and has a high chemical compatibility (Elter, 2019). Print time for each collector was a maximum of 3 hours when printed individually and a fraction of that when several collectors are printed simultaneously. Cost per collector was approximately 3 Euros. As the material can be brittle when the thickness is below 1 mm (Macdonald, 2017), the durability of the 3D printed collector is less than that of one made from stainless steel, but it ensures a smooth surface. This is essential for creating a smooth curtain flow, and at the same time, it decreases the time needed for sample exchange, a topic of central importance for beam-time work at international research facilities as it increases the effective measurement time with “beam on the sample”. The damper is a plastic syringe of 10 mL volume, connected to the fluidic system with a plastic T bridge connector from Carl Roth GmbH. We employed tubing made from chemically resistant tygon (B-44-4X and 2075, purchased from Riesbeck GmbH, Germany) with an inner diameter of 1.6 mm. A microfluidic gear pump (Mikrosysteme GmbH - Germany, model mzr-4622) is employed. This model ensures a large interval of flow, between 0.12 mL/min and 72 mL/min, which can accommodate for a wider set of applications and laser pulses frequencies. The WGJ systems presented here were both operated at a pump speed of 1,500 rpm (0.3 mL/s), the value with the best compromise between noise, stability, and maintaining a kHz shot-to-shot replacement rate.

**FIGURE 2:**
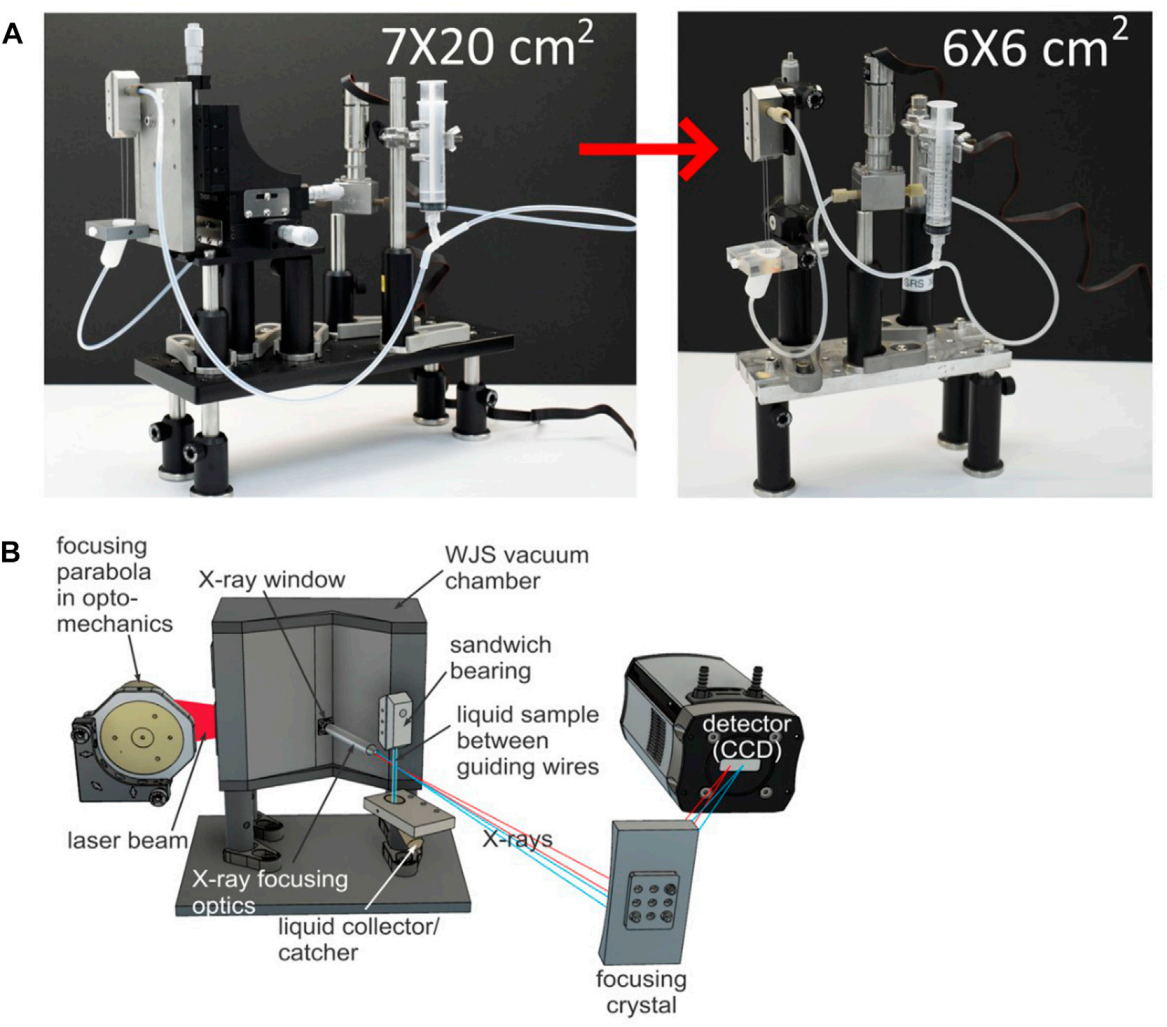
**(A)** Footprint of the earlier version (Picchiotti, 2015) and the greatly reduced footprint of the current one. **(B)** Schematic of the WGJ system within X-ray spectroscopy station at ELI Beamlines.

X-ray set ups are often even more restricted than optical set ups in terms of space available for sample delivery as X-ray is more challenging to re-direct and focus. Figures 2A, B show the water-jet plasma X-ray source under development at ELI Beamlines. When an unfocused X-ray beam is used, a sample needs to be placed as close as possible to the X-ray exit window and consequently it was necessary to reduce the footprint of the WGJ system to increase X-ray intensity on the sample by minimizing the source-to-sample distance. To do so, we removed the L-bearing and attached the jet directly to optical posts through spacers (Thorlabs, Inc.). In the same fashion, the collector has been bolted directly to the same optical post using another set of spacers. In this way, the distance between the port-window and the jet is reduced to a few millimeters. In the case of unfocused X-rays, the beam size is approximately 3 mm in diameter on the sample, thus covering the width of the flat jet, and the alignment to an optical pump-beam for time resolved experiments can be reliably made to a time resolution of the order of 100 fs. Having the option not to use a focusing X-ray element is an important achievement, as this can substantially reduce the time needed to set up the experiment. In the current version, the total footprint of the jet itself has been reduced to a mere 6 × 6 cm^2^, compared to the earlier version (7 × 20 cm^2^), as pictured in the schematics of Figure 2. We note that if an X-ray focusing element (e.g., a polycapillary lens) is used the geometrical constraints relax and the present WGJ system can be placed on a motorized translation stage for precise alignment. The specific WGJ setup and/or X-Ray elements of Figure 2B have been used for experiments published in (Zymaková, 2021; Zymaková, 2022; Zymaková, 2023).

### Wire treatment and wire surface characterization

**FIGURE 3:**
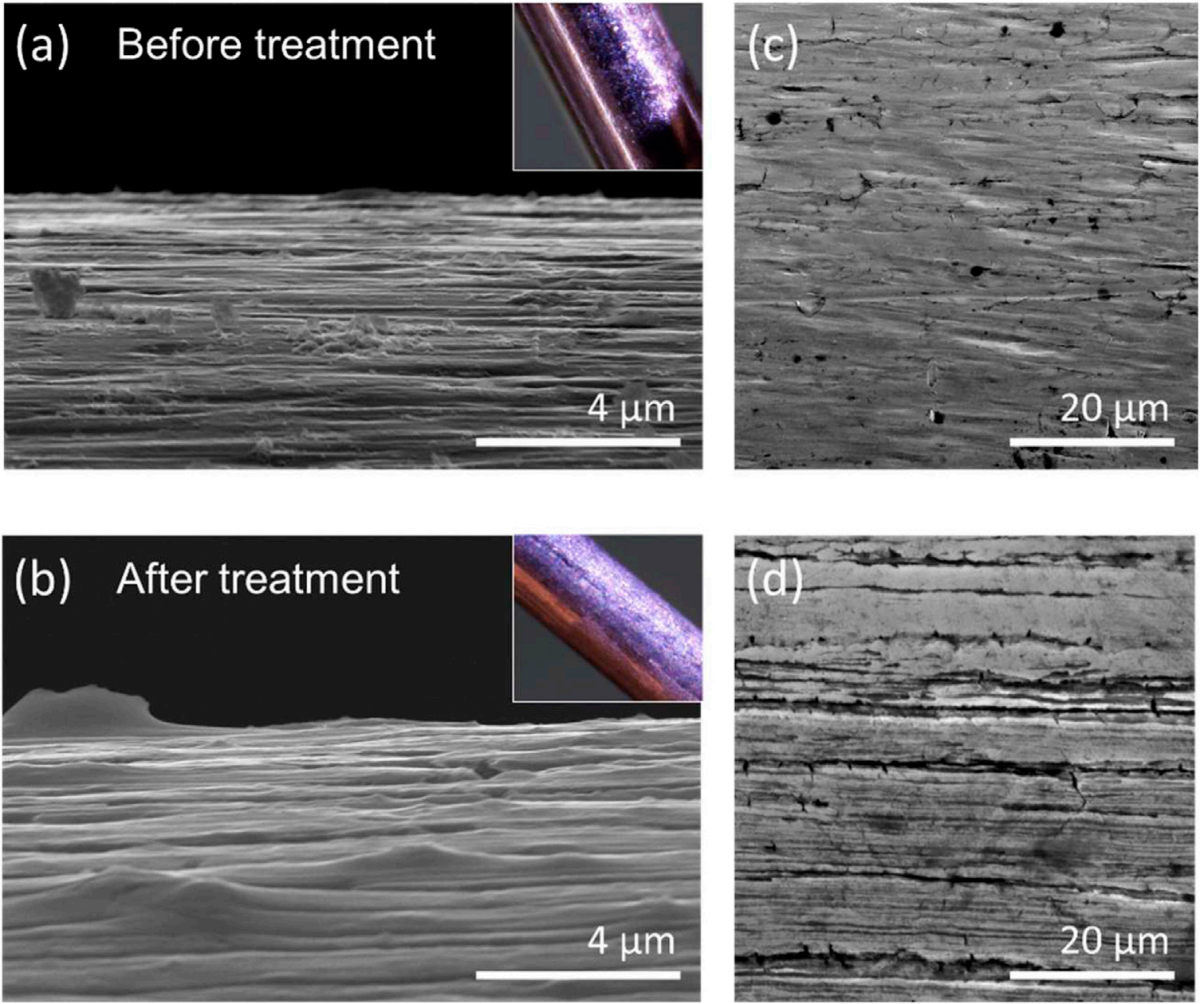
Scanning electron microscope images. Top row shows the untreated wires, bottom row the treated ones. They show details of the edge **(A,B)** and the surface **(C,D)** of the untreated and treated wires respectively,. The inserts of **(A)** and **(B)** are visible microscopy photographs of a detail of the surface of the untreated and treated wires respectively.

Driven by the need to enhance and standardize the optical surface quality at each start of the jet (turning on of the pump), a protocol for chemical treatment (wet etching) of the U-shaped wires was tested. The chemical treatment was intended to sensitize the surface of the wires and increase its wettability, which in turn improved the hands-off character of the creation of the curtain flow, as well as both the long-term stability and the optical quality of the jet when used with a variety of samples of different viscosity. Greater understanding of the mechanisms governing the creation of the film, and the surface interfaces between the wires and the liquid, will increase control of the thickness of the liquid jet and therefore signal-to-noise ratio in experiments. A selection of SEM images of wire surface details is shown in Figure 3. In (a) the surface of the untreated wires can be seen from the top, in (b) of the treated ones, and in (c) and (d) SEM images with higher magnification of the edges of the untreated and treated wires respectively. Scalebars are 20 μm and 4 μm, as stated in the images, respectively. The inserts on the top left are images from an optical microscope. An initial hypothesis was that the wettability increased, and that this was due to chemical changes of the wire surface due to the formation of a potentially smoother oxidized layer, as can be observed in the magnified optical microscopy photos in the inserts in Figures 3A, B. While it is correct that the first layers of the stainless-steel surface are oxidized after the hydrochloric treatment, demonstrated by the change of color (grayer) and loss of shine of the surface of the wires, as well as by the reddish color of the hydrochloric acid used for the wire treatment, there is also a profound change of the surface geometry at the micro-level, as seen in the SEM images of Figure 3 and as we show later in this publication by the contact angle measurements. In fact, the wettability does not increase after the treatment, and the wires become more hydrophobic instead.

The series of micro-grooves along the length of the wires, already present in the untreated wires, is increased in regularity by the hydrochloric acid treatment. In the edge SEM images of Figure 3 bottom row, there appears to be no change in the dimensions of the channels, although a more in-depth study is required to confirm this. The difference between the two systems is mainly in the homogeneous distribution of the channels observed after treatment, causing the patches (or domains) of roughness on the surface to differ less.

The fluidic properties are associated with certain structures to the surface geometry at the micro-level. In particular, by increasing the roughness according to the uniform distribution brought forward by treatment (the etching process). Indeed, also nature hosts such phenomena. Lotus leaves have micro- and nano-structured surfaces composed of 10–15 μm sized papillae which, together with epicuticular waxes superimposed to this structure, cause the leaf surface to be ultra-hydrophobic (Barthlott, 1997). In general, the effect of micro-structuring a surface to make it hydrophobic or super-hydrophobic is very well known in the micro- and nano-fabrication community (Dong, 2019; Wang, 2022).

**FIGURE 4:**
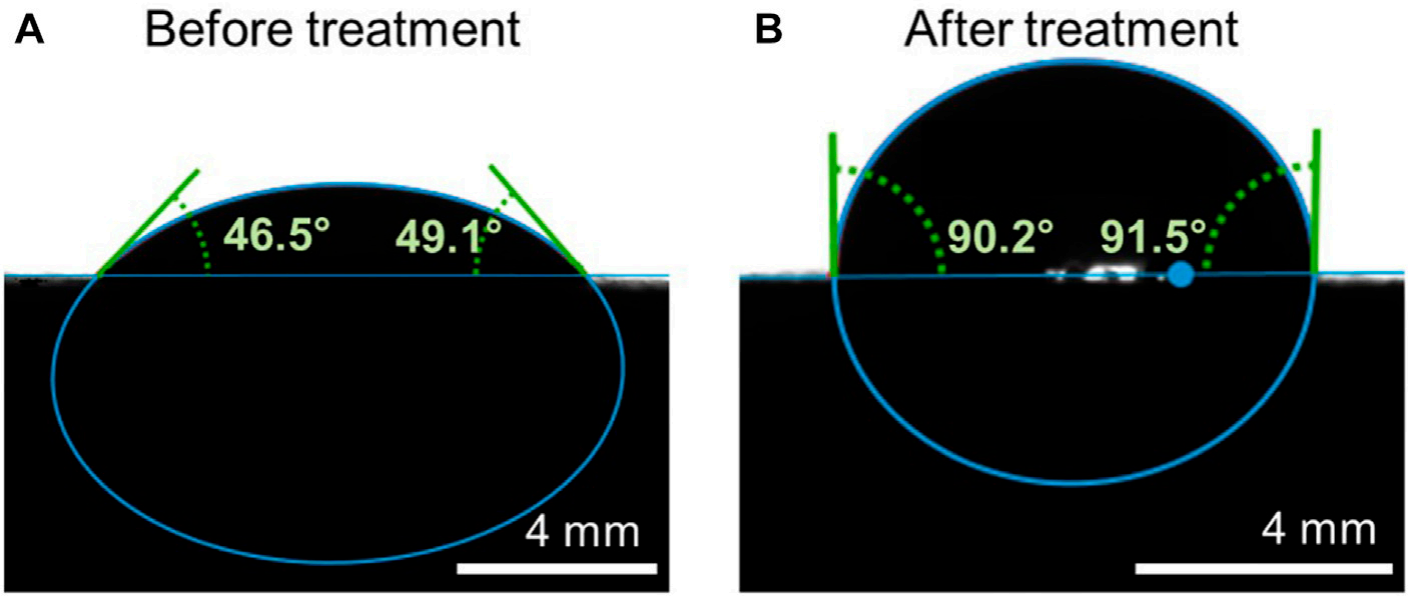
Contact angle measurements from a flat piece of 316 stainless steel. **(A)**: before wet-etching; **(B)**: after 30 min of wet-etching with HCl.

**FIGURE 5:**
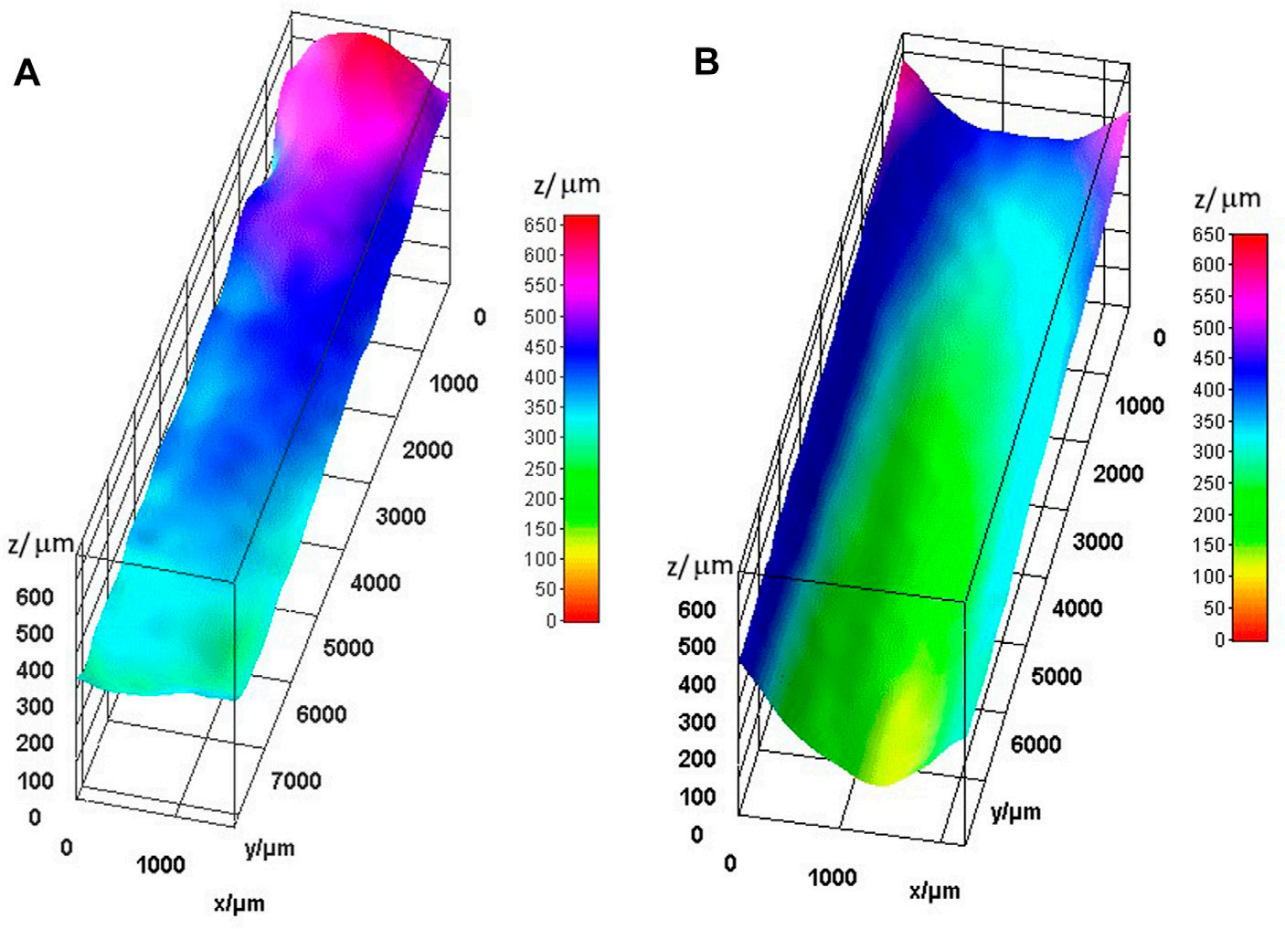
Difference on the optical quality of fluorescence light emitted from the WGJ, filled with a Rhodamine 6G solution, before **(A)** and after **(B)** hydrochloric acid treatment of the U-shaped wires.

To verify that the wet etching used to modify the wire surfaceyields an increased contact angle, hence a decreased wettability, the same protocol as used for the wires was applied to a larger, rectangular piece of the same stainless steel (316) material, with a flat surface. The choice of a piece with a flat surface is guided by the fact that the wires have too small of a diameter (0.5 mm) to be able to perform contact angle measurements. Following the treatment, the water contact angle (WCA) on the treated and un-treated surfaces was measured and compared. In Figure 4, we show that the WCA from the stainless steel, before and after 30 min of wet etching, increases from approximately 47.8°–90.85°, confirming a substantial increase of contact angle (almost double) with the treatment proposed in the present publication. It is then clear, both from the data presented here and the literature (Yang, 2011; Li, 2012; Zhang, 2022), that the increased roughness of the surface, increases the WCA, for these treatment protocols. From the combination of information from the SEM images in Figure 3, the WCA values in Figure 4 and the fluorescence images of Figure 5, we conclude that the roughness of the wires plays a key role in the resulting surface topography of the liquid sheet. Specifically, we tentatively suggest that the increase in regularity of the channels in the wires surface causes the observed regularization of the liquid surface, and emphasize that this effect on the interface between the wires surface and the liquid, is present as far away as a few millimeters from the surface of the wire. The optimization of the treatment protocol, which will be the subject for further work, will provide the possibility to control the topological regularity and thickness of the liquid employed in the WGJ.

The main advantage of having a smooth liquid film is that the quality of the measured signal will be optimal, since the distortion of the beam profile and unwanted scattering of the emitted signal will be minimized. Figure 1 shows two images of the two jets with untreated (a) and treated (b) wires. There, the difference between the two liquid sheets is already visible. In the case of the non-treated wires, visible optical disturbances created by the turbulences and irregularities in the liquid surface are observable, while this is not the case in the one with the treated wires. We observed this difference also in a direct experiment. A laser beam was shone through the liquid of each system, and the beam profile of the transmitted beam was projected on a white screen behind the sheet. The projections of the beams can be seen in Figure 1. From the results we deduced that the hydrochloric acid treatment of the wires qualitatively improves the homogeneity of the light transmitted though the liquid film: the left image, taken using the system with untreated wires, shows substantial distortions and aberrations in the transmitted beam, while the image to the right, taken with the system with treated wires, shows a uniform distribution of the transmitted light. The elongation of the beam on the screen is caused by the size of the laser beam. Since the diameter of the laser beam was larger than the dimension of the liquid sheet, scattering from the vertical walls of the wires created the characteristic elongated shape of light on the screen in Figure 1 (right).

### Optical characterization of the liquid flow sheet

In a more detailed experiment, we measured fluorescence to quantitatively assess the optical surface quality, as it is done for the characterization for jetting systems with mixing microchips produced by soft lithography (Hejazian, 2020) and adapted the characterization to both the WGJ systems used for the previous qualitative experiment. The results are presented in Figure 5 after normalization and scaling, as described in Materials and Methods. The difference between the optical surface quality formed with WGJ systems mounting treated and untreated wires shown in Figures 1, 5 respectively, is quite substantial. Specifically, in Figure 5 one can see the improvement in regularity and smoothness of the liquid surface resulting from treating the wires with hydrochloric acid, resulting in a substantial improvement of the optical quality. One can also notice that the top part has a smaller variation along the *X*-axis, hence the best optical quality is a few millimeters below the exit of the sandwich bearings. The thickness of the liquid layer at the center of the jet, as determined by this method, is between 300 μm and 600 μm for the WGJ with untreated wires and between 300 μm and 150 μm for the jet produced with the treaded wires. This supports our recommendation to perform the measurements with a focused beam at the best position, which is the center between the wires and few millimeters below the end of the sandwich bearing. There the thickness of the jet at the equivalent flow rate of 0.15 mL/s was measured to be 150 μm. The measurements reported in this study show a thickness between 250 μm and 150 μm in the equivalent position in the present jet, confirming that the two methods yield similar results, but with the added value of the fluorescence method, that it provides a value of the thickness of all the liquid layer between the wires at the simultaneously, resulting in a more complete set of data collected at the same time.

It is known from the literature that to change the wettability of steel, one must act both through lowering the surface energy and increasing the roughness of the surface (Yu, 2013). Wettability can be tuned by means of chemical and/or physical treatments, and in case of stainless steel, hydrochloric acid has been employed to change wettability (Yang, 2011). In particular, etching of stainless steel 316 with hydrochloric acid, very similar to the treatment done in this study, has been shown to change wettability (Li, 2012). In this study, stainless steel 316 material etched with hydrofluoric acid showed an increased mean roughness depending on etching time with a saturation point after 30 min, but a decreased contact angle, hence the opposite result as the one presented here. Other works show that surface micro-structures leads to a significant change in wettability for droplets and static systems (Rauscher, 2008a), for rivulets (Wilson, 2012; Monnier, 2010), as well as stainless steel and lithium systems (Szott, 2016). Finally, a very recent publication (Zhang, 2022) describes a protocol to increase the contact angle (thus modifying wettability) of stainless-steel surfaces by the combined action of hydrochloric acid and ferric chloride solutions, followed by fluoroalkyl silane. In this work the increased contact angle is linked to the increase of roughness of the surface until a maximum hydrophobic effect, to then decrease again, although their CA measurements were conducted after fluoroalkyl silane modification. Further studies are already ongoing in our laboratories, to clarify the exact mechanism that allows us to increase the CA of 316 L stainless steel by hydrochloric acid wet etching.

For the wires in the present work, the presence of long and narrow channels along their length (both untreated and treated) is directly connected to how the wires are produced (Bourauel, 1998). For wires with small diameters, the chosen method is wet drawing (Sadevinox). This method tends to apply shear forces along the length of the wires as they are pulled through the various machinery parts, creating an underlying structure that that the hydrochloric acid treatment eventually uncovers, making the channel depths more pronounced and more homogeneously distributed along the wire surface.

As a result of the wet etching the channels at the wire surface become more regular and the wires display fewer patches with different roughness. We hypothesize that this effect of the wet etching modifies the wettability properties of the wires resulting, in turn, in controllable effects on the formation of the liquid jet. Comparing the two images in Figure 5 we clearly see that the wet etching significantly reduces the roughness of the liquid surface generated by the WGJ. In the right image, when treated wires are used, the roughness of the liquid surface is substantially decreased, and the topology of the liquid surface is smoother, compared to the left image of Figure 5, where untreated wires were employed. We link this development to an increase in the wettability induced by the wet etching.

## Conclusion

This work describes developments to a liquid jet system (WGJ) that most notably provide a compact footprint of just 6 × 6 cm^2^, and a more homogeneous liquid surface yielded by wet etching of the wires guiding the liquid. The reduced footprint (almost ×4 smaller than our previously reported jet) allows it to fit into much smaller spaces and workstations, including the X-ray spectroscopy station at the ELI beamlines facility, thus meeting the requirements of more demanding setups. The system additionally incorporates a number of 3D printed parts that have been both successfully tested and extensively used. The use of 3D printed parts significantly reduces production time and cost while reducing risk of chemical cross-contamination when operated at user facilities where frequent sample exchanges are required and access to beam time is limited. A future development plans include printing and testing the sandwich block using the same polymeric material as the collector.

We also present a protocol for wet etching of the U-shaped stainless-steel wires used between the sandwich block in the WGJ system. Wet-etching substantially changes the surface of the stainless steel and the contact angle, thus improving the creation and the overall optical quality of the jet film between the wires, and significantly improves the reliability of the system. The hydrochloric acid reacts with the surface of the wires bringing forth long and narrow channels, and in general increasing roughness density, rather than smoothing the stainless-steel surface. Indeed, the core finding of the present study is the ability to modify the topological shape of the liquid surface by modifying the surface of the wires at the micrometer-scale, which affects the contact angle and wettability, ultimately changing the surface quality of the liquid millimeters away from the surfaces of the wires. In this way, we can control the topology of a free standing, but moving, layer of liquid by tweaking the surface roughness of the (stainless-steel) wires.

To characterize the improved WGJ system, we have developed and used a relatively simple and inexpensive fluorescence imaging methodology, together with SEM images of the wires surfaces. This work represents, to our knowledge, the first complete characterization of the surface of wettable parts, together with the topological surface and thickness of the liquid in a macroscopic (millimeters to centimeters) low-pressure flow-system. The fluorescence characterization is also suitable to test, characterize and optimize jet systems just before they are employed in complex optical and X-ray spectroscopy setups decreasing the time needed for online optimization at the facilities or during experimental time.

In conclusion, we presented here a low-pressure flow jet system (WGJ) with small foot-print, very small liquid sample consumption, simple and in-expensive to build, and a fluorescence-based optical method to characterize qualitatively and quantitatively the thickness of the liquid sheet and its surface topology. While we do not expect that the WGJ replaces other existing technologies in the flow-jet landscape, we envisage that it fills a vacuum, where high-pressure jets do not work for biologically relevant and expensive samples, samples that clog easily small noozles, or where experimental space is limited.

## Data Availability

The raw data supporting the conclusion of this article will be made available by the authors, without undue reservation.

## References

[B2] Barnard J. C. T. Lee J. P. Alexander O. Jarosch S. Garratt D. Picciuto R. (2022). Delivery of stable ultra-thin liquid sheets in vacuum for biochemical spectroscopy. Front. Mol. Biosci. 9, 1044610–1044619. 10.3389/fmolb.2022.1044610 36452452 PMC9701818

[B3] Barthlott W. Neinhuis C. (1997). Purity of the sacred lotus, or escape from contamination in biological surfaces. Planta 202 (1), 1–8. 10.1007/s004250050096

[B4] Bourauel C. Fries T. Drescher D. Plietsch R. (1998). Surface roughness of orthodontic wires via atomic force microscopy, laser specular reflectance, and profilometry. Eur. J. Orthod. 20 (1), 79–92. 10.1093/ejo/20.1.79 9558768

[B5] Cannelli O. Bacellar C. Ingle R. A. Bohinc R. Kinschel D. Bauer B. (2019). Toward time-resolved laser T-jump/X-ray probe spectroscopy in aqueous solutions. Struct. Dyn. 6 (6), 064303. 10.1063/1.5129626 31832487 PMC6906120

[B6] Chapman H. N. Caleman C. Timneanu N. (2014). Diffraction before destruction. Philosophical Trans. R. Soc. B Biol. Sci. 369 (1647), 20130313. 10.1098/rstb.2013.0313 PMC4052855 24914146

[B7] Dong L. Zhang Z. Ding R. Wang L. Liu M. Weng Z. (2019). Controllable superhydrophobic surfaces with tunable adhesion fabricated by laser interference lithography. Surf. Coatings Technol. 372, 434–441. 10.1016/j.surfcoat.2019.05.039

[B8] Duris J. Li S. Driver T. Champenois E. G. MacArthur J. P. Lutman A. A. (2020). Tunable isolated attosecond X-ray pulses with gigawatt peak power from a free-electron laser. Nat. Photonics 14 (1), 30–36. 10.1038/s41566-019-0549-5

[B9] Elter A. Dorsch S. Mann P. Runz A. Johnen W. Karger C. P. (2019). Compatibility of 3D printing materials and printing techniques with PAGAT gel dosimetry. Phys. Med. Biol. 64 (4), 04NT02. 10.1088/1361-6560/aafef0 30650389

[B10] Fondell M. Eckert S. Jay R. M. Weniger C. Quevedo W. Niskanen J. (2017). Time-resolved soft X-ray absorption spectroscopy in transmission mode on liquids at MHz repetition rates. Struct. Dyn. 4 (5), 054902. 10.1063/1.4993755 28852689 PMC5555770

[B11] Galestian Pour A. Lincoln C. N. Perlík V. Šanda F. Hauer J. (2017). Anharmonic vibrational effects in linear and two-dimensional electronic spectra. Phys. Chem. Chem. Phys. 19 (36), 24752–24760. 10.1039/c7cp05189a 28868559

[B12] Galinis G. Strucka J. Barnard J. C. T. Braun A. Smith R. A. Marangos J. P. (2017). Micrometer-thickness liquid sheet jets flowing in vacuum. Rev. Sci. Instrum. 88 (8), 083117. 10.1063/1.4990130 28863712

[B13] Grünbein M. L. Nass Kovacs G. (2019). Sample delivery for serial crystallography at free-electron lasers and synchrotrons. Acta Crystallogr. Sect. D. Struct. Biol. 75 (2), 178–191. 10.1107/S205979831801567X 30821706 PMC6400261

[B14] Hejazian M. Darmanin C. Balaur E. Abbey B. (2020). Mixing and jetting analysis using continuous flow microfluidic sample delivery devices. RSC Adv. 10 (27), 15694–15701. 10.1039/D0RA00232A 35493684 PMC9052392

[B15] Kim J. Kim K. H. Oang K. Y. Lee J. H. Hong K. Cho H. (2016). Tracking reaction dynamics in solution by pump–probe X-ray absorption spectroscopy and X-ray liquidography (solution scattering). Chem. Commun. 52 (19), 3734–3749. 10.1039/C5CC08949B 26785280

[B16] Laimgruber S. Schachenmayr H. Schmidt B. Zinth W. Gilch P. (2006). A femtosecond stimulated Raman spectrograph for the near ultraviolet. Appl. Phys. B 85 (4), 557–564. 10.1007/s00340-006-2386-8

[B17] Li L. Breedveld V. Hess D. W. (2012). Creation of superhydrophobic stainless steel surfaces by acid treatments and hydrophobic film deposition. ACS Appl. Mater. Interfaces 4 (9), 4549–4556. 10.1021/am301666c 22913317

[B18] Macdonald N. P. Cabot J. M. Smejkal P. Guijt R. M. Paull B. Breadmore M. C. (2017). Comparing microfluidic performance of three-dimensional (3D) printing platforms. Anal. Chem. 89 (7), 3858–3866. 10.1021/acs.analchem.7b00136 28281349

[B19] Menzi S. Knopp G. Al Haddad A. Augustin S. Borca C. Gashi D. (2020). Generation and simple characterization of flat, liquid jets. Rev. Sci. Instrum. 91 (10), 105109. 10.1063/5.0007228 33138597

[B20] Miaja-Avila L. O’Neil G. C. Uhlig J. Cromer C. L. Dowell M. L. Jimenez R. (2015). Laser plasma x-ray source for ultrafast time-resolved x-ray absorption spectroscopy. Struct. Dyn. 2 (2), 024301. 10.1063/1.4913585 26798792 PMC4711629

[B21] Mikrosysteme GmbH (2020). Hnp Mikrosysteme GmbH mzr-4622 data sheet. https://www.hnp-mikrosysteme.de/de/produkte/detail/mzr-4622-1/pdf/.

[B22] Monnier H. Mhiri N. Falk L. (2010). Falling liquid film stability in microgas/liquid absorption. Chem. Eng. Process. Process Intensif. 49 (9), 953–957. 10.1016/j.cep.2010.05.001

[B23] Oppermann M. Bauer B. Rossi T. Zinna F. Helbing J. Lacour J. (2019). Ultrafast broadband circular dichroism in the deep ultraviolet. Optica 6 (1), 56. 10.1364/optica.6.000056

[B24] Pastorczak M. Nejbauer M. Radzewicz C. (2019). Femtosecond infrared pump-stimulated Raman probe spectroscopy: The first application of the method to studies of vibrational relaxation pathways in the liquid HDO/D2O system. Phys. Chem. Chem. Phys. 21 (31), 16895–16904. 10.1039/c9cp00855a 31215570

[B25] Penzkofer A. Leupacher W. (1987). Fluorescence behaviour of highly concentrated rhodamine 6G solutions. J. Luminescence 37 (2), 61–72. 10.1016/0022-2313(87)90167-0

[B26] Picchiotti A. Nenov A. Giussani A. Prokhorenko V. I. Miller R. J. D. Mukamel S. (2019). Pyrene, a test case for deep-ultraviolet molecular photophysics. J. Phys. Chem. Lett. 10 (12), 3481–3487. 10.1021/acs.jpclett.9b01325 31081636 PMC6774270

[B27] Picchiotti A. Prokhorenko V. I. Miller R. J. D. D. (2015). A closed-loop pump-driven wire-guided flow jet for ultrafast spectroscopy of liquid samples. Rev. Sci. Instrum. 86 (9), 093105. 10.1063/1.4929860 26429427

[B28] Prokhorenko V. I. Picchiotti A. Pola M. Dijkstra A. G. Miller R. J. D. D. (2016). New insights into the photophysics of DNA nucleobases. J. Phys. Chem. Lett. 7 (22), 4445–4450. 10.1021/acs.jpclett.6b02085 27786479

[B29] Rauscher M. Dietrich S. (2008). Wetting phenomena in nanofluidics. Annu. Rev. Mater. Res. 38 (1), 143–172. 10.1146/annurev.matsci.38.060407.132451

[B30] Rimington R. P. Capel A. J. Player D. J. Bibb R. J. Christie S. D. R. Lewis M. P. (2018). Feasibility and biocompatibility of 3D-printed photopolymerized and laser sintered polymers for neuronal, myogenic, and hepatic cell types. Macromol. Biosci. 18 (7), 1800113. 10.1002/mabi.201800113 29900676

[B31] Sadevinox (2017). Stainless steel wire. https://www.stainlesssteelwire.com/stainless-steel-wire-history-manufacturing-applications.html.

[B32] Schreck S. Gavrila G. Weniger C. Wernet P. (2011). A sample holder for soft x-ray absorption spectroscopy of liquids in transmission mode. Rev. Sci. Instrum. 82 (10), 103101. 10.1063/1.3644192 22047274

[B33] Stange U. C. Temps F. (2018). Ultrafast electronic deactivation of UV-excited adenine and its ribo- and deoxyribonucleosides and -nucleotides: A comparative study. Chem. Phys. 515, 441–451. 10.1016/j.chemphys.2018.08.031

[B34] Stratasys Ltd (2020). Vero clear:rigid transparent 3D print-ing material.

[B35] Szlachetko J. Nachtegaal M. de Boni E. Willimann M. Safonova O. Sa J. (2012). A von Hamos x-ray spectrometer based on a segmented-type diffraction crystal for single-shot x-ray emission spectroscopy and time-resolved resonant inelastic x-ray scattering studies. Rev. Sci. Instrum. 83 (10), 103105. 10.1063/1.4756691 23126749

[B36] Szott M. Fiflis P. Kalathiparambil K. Shchelkanov I. Ruzic D. N. Jurczyk B. (2015). “Wetting of lithium on nanostructured surfaces for first wall components,” in Proceedings of the 2015 IEEE 26th Symposium on Fusion Engineering (SOFE), Austin, TX, USA, 2016-May, 1–4. 10.1109/SOFE.2015.7482285

[B37] Tauber M. J. Mathies R. A. Chen X. Bradforth S. E. (2003). Flowing liquid sample jet for resonance Raman and ultrafast optical spectroscopy. Rev. Sci. Instrum., 74(11), 4958. 4960. 10.1063/1.1614874

[B38] Wang Z. Paul S. Stein L. H. Salemi A. Mitra S. (2022). Recent developments in blood-compatible superhydrophobic surfaces. Polymers 14 (6), 1075. 10.3390/polym14061075 35335407 PMC8953528

[B39] Weierstall U. (2014). Liquid sample delivery techniques for serial femtosecond crystallography. Philosophical Trans. R. Soc. B Biol. Sci. 369 (1647), 20130337–7. 10.1098/rstb.2013.0337 PMC4052872 24914163

[B40] Wilson D. I. Le B. L. Dao H. D. A. Lai K. Y. Morison K. R. Davidson J. F. (2012). Surface flow and drainage films created by horizontal impinging liquid jets. Chem. Eng. Sci. 68 (1), 449–460. 10.1016/j.ces.2011.10.003

[B41] Yu S. Wang X. Wang W. Yao Q. Xu J. Xiong W. (2013). A new method for preparing bionic multi scale superhydrophobic functional surface on X70 pipeline steel. Appl. Surf. Sci. 271, 149–155. 10.1016/j.apsusc.2013.01.152

[B42] Zhang Y. Zhang Z. Yang J. Yue Y. Zhang H. (2022). Fabrication of superhydrophobic surface on stainless steel by two-step chemical etching. Chem. Phys. Lett. 797, 139567. 10.1016/j.cplett.2022.139567

[B44] Zymaková A. Khakurel K. Picchiotti A. Błachucki W. Szlachetko J. Rebarz M. (2020). Implementation of a crossed-slit system for fast alignment of sealed polycapillary X-ray optics. J. Synchrotron Radiat. 27 (6), 1730–1733. 10.1107/S1600577520012217 33147201 PMC7642965

[B43] Zymaková A. Albrecht M. Antipenkov R. Špaček A. Karatodorov S. Hort O. (2021). First experiments with a water-jet plasma X-ray source driven by the novel high-power–high-repetition rate L1 Allegra laser at ELI Beamlines. J. Synchrotron Radiat. 28 (6), 1778–1785. 10.1107/S1600577521008729 34738931 PMC8570212

[B1] Zymaková A., Precek M., Picchiotti A., Błachucki W., Szlachetko J., Vankó G. (2022). In preparation.

[B45] Zymaková A. Kantarelou V. Stanček S. Bursak D. Danielisová A. Anagnostopoulos D. F. (2023). A fast-integrated x-ray emission spectrometer dedicated to the investigation of Pt presence in gold Celtic coins (3rd–1st century BCE). X-ray spectrom., 1–11. 10.1002/xrs.3354

